# Epileptic Encephalopathy After Human Herpes Virus 6-Related Post-Transplant Acute Limbic Encephalitis in Children: A Case Report and Review of the Literature

**DOI:** 10.7759/cureus.84647

**Published:** 2025-05-22

**Authors:** Yusuke Goto, Yusuke Takezawa, Saori Katayama, Yurika Numata-Uematsu, Mitsugu Uematsu

**Affiliations:** 1 Department of Pediatrics, Tohoku University School of Medicine, Sendai, JPN

**Keywords:** electroencephalographic, epilepsy, epileptic encephalopathy, epileptic spasm, human herpes virus 6, post-transplant acute limbic encephalitis

## Abstract

Post-transplant human herpes virus 6 (HHV6) encephalitis can be followed by refractory epilepsy accompanied by intellectual decline after several months. However, such cases are extremely rare, and the disease mechanism remains elusive. We present the case of an eight-year-old boy who presented with epileptic encephalopathy 11 months after developing post-transplant acute limbic encephalitis (PALE) caused by HHV6. The patient developed multiple types of seizures, primarily characterized by epileptic spasms. Significant electroencephalographic (EEG) abnormalities were noted during the interictal period, along with regression of cognitive and language functions and progressive atrophy of the entire brain, including the hippocampus. He was managed with multiple antiepileptic drugs, although his seizures remained uncontrolled for one year after epilepsy onset. Herein, we summarized and analyzed the clinical features of the previously reported cases and the present case. The median time from the onset of HHV6 PALE to epilepsy was 11.5 months. Developmental regression or cognitive decline, multiple seizure types including tonic seizures, generalized slow waves, multifocal spike-wave activity on interictal EEG, brain changes such as hippocampal sclerosis, and poor seizure prognosis are common features. The disease was classified as epileptic encephalopathy following HHV6-related PALE (EE-PALE). This case not only provides additional evidence that EE-PALE is a distinct disease with consistent clinical features but is also expected to contribute to the identification of its pathogenesis and effective treatment.

## Introduction

Human herpes virus 6 (HHV6) is typically acquired during infancy and causes a febrile illness that is sometimes accompanied by exanthema subitum or febrile convulsions. After primary infection, HHV6 becomes latent. Reactivation of HHV6 mainly causes post-transplant acute limbic encephalitis (PALE) during latent infection [1]. Patients with PALE experience confusion, anterograde amnesia, syndrome of inappropriate antidiuretic hormone secretion (SIADH), and clinical seizures that manifest acutely 29 days after transplantation. However, seizures are typically controlled by treatment with one or two antiepileptic drugs [1,2]. Antiviral drugs, including ganciclovir, are reportedly effective against HHV6 PALE [3].

A handful of case reports have focused on children who developed intractable epilepsy within a certain period after the onset of HHV6 PALE [4-6]. Patients with this disease share common characteristics, such as various types of seizures including tonic seizures, cognitive decline and developmental regression, language and memory impairment, atrophy or sclerosis of the hippocampus, and poor response to antiepileptic drugs and immunomodulatory therapy. No evidence of chronic progressive inflammation or secondary immune activation was identified on cerebrospinal fluid examinations or magnetic resonance imaging (MRI) scans; therefore, Maizuru et al. defined this condition as epileptic encephalopathy following HHV6 PALE [6].

The patient described in this case report developed intractable epilepsy following HHV6 PALE and demonstrated various types of seizures, significant electroencephalographic (EEG) abnormalities, brain atrophy involving the hippocampus on one side, and cognitive decline. Referring to previous reports of similar cases, we summarized the clinical features of this disease. Therefore, we aimed to provide additional evidence to understand and characterize this rare disease.

## Case presentation

**Figure 1:**
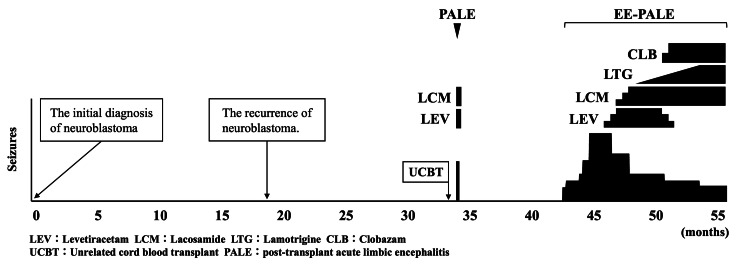
The patient’s treatment progress The initial diagnosis of neuroblastoma was made on day 0. The patient relapsed 18 months later and underwent cord blood transplantation 33 months after initial diagnosis. The patient developed PALE caused by the HHV6 22 days after transplantation. He further developed acute symptomatic seizures, which improved with short-term LEV and LCM. After a seizure-free period, nine months post-transplantation, he developed epileptic spasms and tonic seizures in his upper limbs, and his cognitive function gradually declined. Twelve months after the transplantation, the patient was diagnosed with EE-PALE based on video electroencephalographic findings, and LEV treatment was resumed. With the addition of LCM, lamotrigine, and clobazam, the frequency of seizures decreased, but persisted for one year after disease onset. MRI performed 18 months after transplantation revealed brain atrophy, mainly in the right hippocampus. EE-PALE, epileptic encephalopathy following HHV6-related PALE; HHV6, human herpes virus 6; LCM, lacosamide; LEV, levetiracetam; MRI, magnetic resonance imaging; PALE, post-transplant acute limbic encephalitis

**Figure 2:**
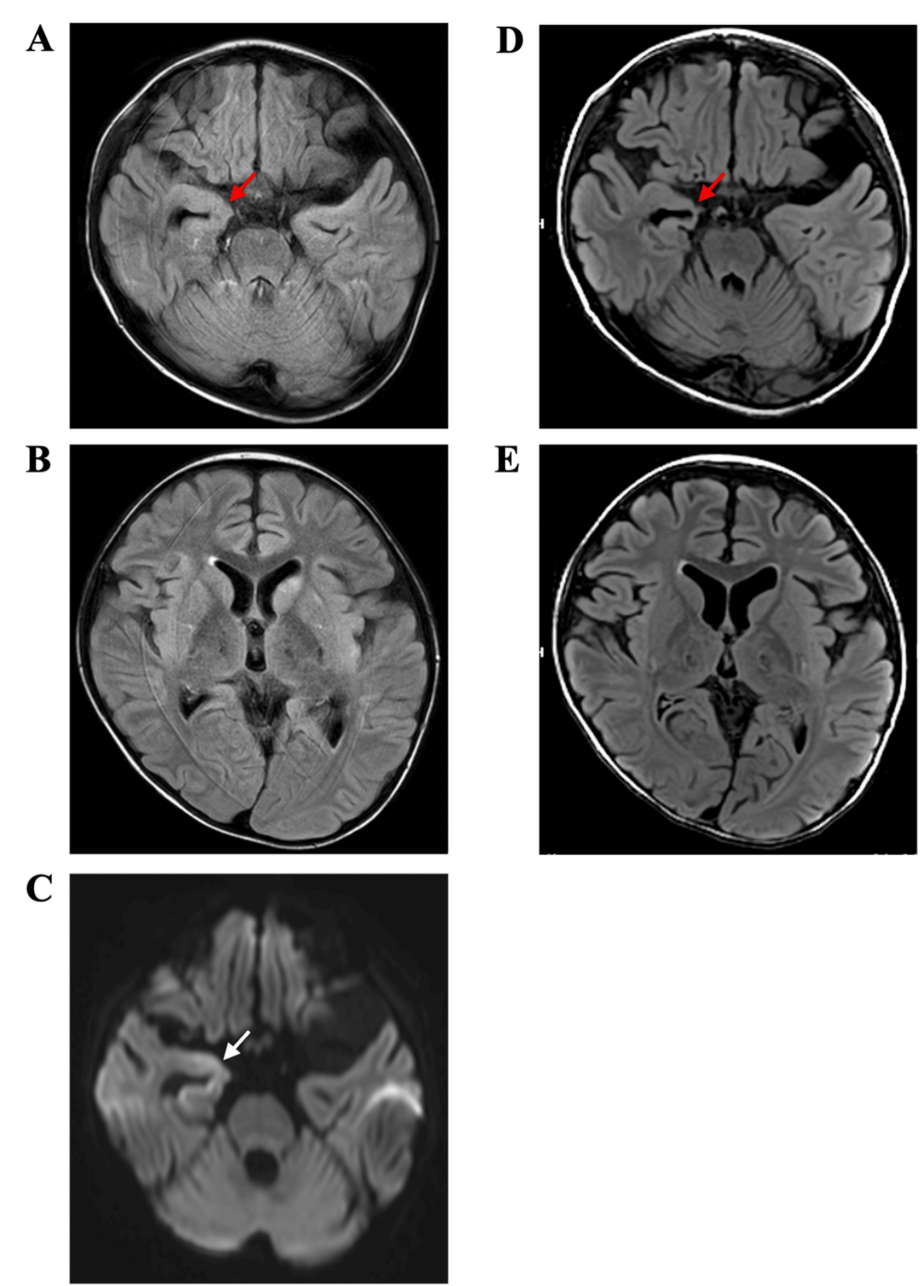
MRI of the patient's brain (A–C) Brain magnetic resonance images obtained in the acute phase after the onset of PALE 22 days after transplantation. (A, B) FLAIR images. (C) Diffusion-weighted image showing a high-signal area in the right hippocampus (white arrow). (D, E) FLAIR magnetic resonance images of the patient's brain acquired 17 months after the onset of PALE. Atrophy of the entire brain, mainly the right hippocampus (red arrow), is noted. FLAIR, fluid-attenuated inversion recovery; MRI, magnetic resonance imaging; PALE, post-transplant acute limbic encephalitis

An eight-year-old boy presented to our hospital with epilepsy and cognitive decline (Figure 1). At five years of age, he was diagnosed with an intra-abdominal primary neuroblastoma that had metastasized to the bones and distant lymph nodes. As a high-risk patient, he underwent multimodal therapy in accordance with the JN-H-15 protocol of the Japan Neuroblastoma Study Group (JNBSG) as a high-risk patient [7]. Induction therapy was administered, followed by pre-transplant therapy and autologous peripheral blood stem cell transplantation. Subsequently, surgical resection of the primary tumor, radiation therapy at the primary tumor site, and differentiation therapy with 13-cis-retinoic acid were performed. At six years of age, the patient relapsed owing to multiple bone metastases and was administered combination therapy with irinotecan, temozolomide, and anti-GD2 antibody, followed by umbilical cord blood transplantation. Tacrolimus (administered for four months from the day before transplantation) and short-term methotrexate were administered as graft-versus-host disease prophylaxis. Eight days post-transplantation, the patient developed fever and was treated with antibiotics for bacteremia. Since his fever persisted even after the bacteremia had resolved, we administered methylprednisolone (from days 14 to 70 after transplantation, maximum dose of 1.5 mg/kg/day) as a treatment for engraftment syndrome. The patient developed seizures 22 days after transplantation, and HHV6 was detected using the FilmArray Meningitis/Encephalitis (ME) panel in the cerebrospinal fluid. A high-signal area was observed in the right medial temporal lobe on diffusion-weighted MRI of the brain (Figures 2A-2C), leading to the diagnosis of HHV6 PALE. After antiviral therapy with foscarnet sodium hydrate and antiseizure therapy with levetiracetam (LEV) and lacosamide (LCM), the patient’s clinical symptoms resolved. Since the epileptic EEG abnormalities disappeared in the short term, we diagnosed these seizures as acute symptomatic seizures and discontinued anti-seizure therapy after nine days. However, a hematoma formed around the conus medullaris and cauda equina after lumbar puncture, resulting in the incomplete paralysis of both lower limbs and bladder-rectum dysfunction due to nerve compression. Nine months post-transplant, he began to experience sudden and brief muscle contractions in the limbs and trunk, as well as stiffness in the upper limbs for several seconds, both of which gradually became more frequent. During the same period, the patient exhibited progressive cognitive decline and speech disorders. He visited our hospital 12 months after the transplant and was admitted for a detailed examination for epilepsy.

**Figure 3:**
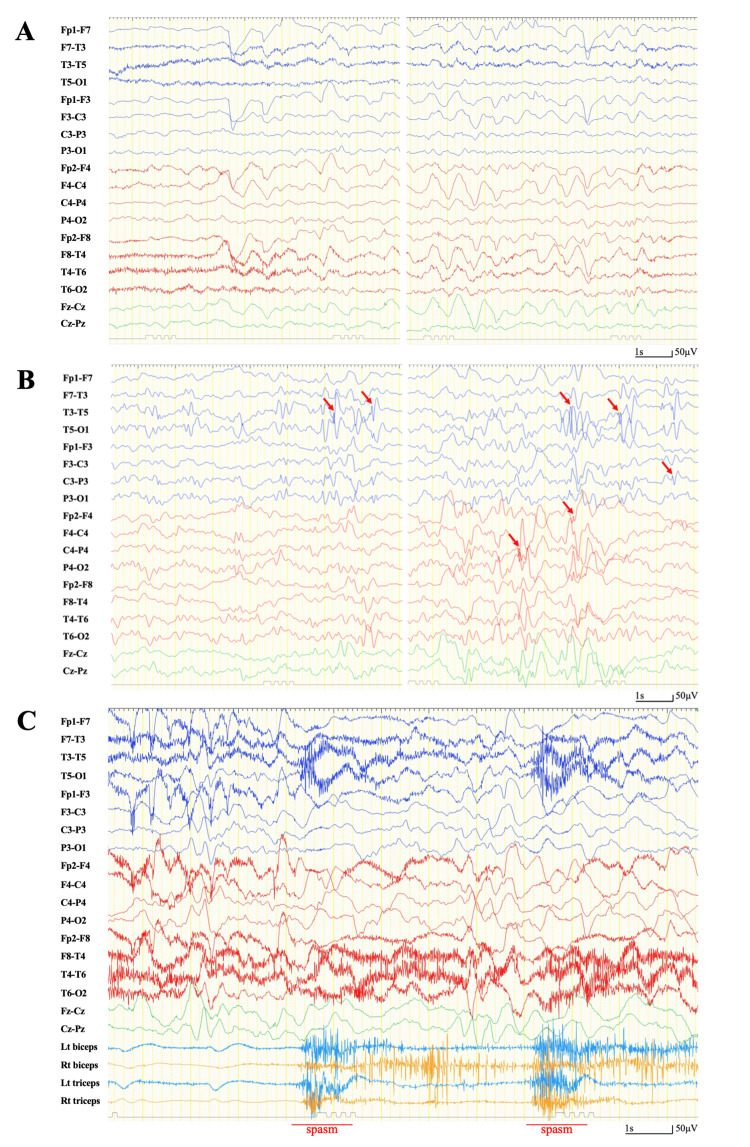
vEEG findings Around 11 months after the onset of PALE, a vEEG was recorded using a longitudinal bipolar montage (also known as a double banana montage) with a sensitivity of 10 μV, a high-frequency filter set at 60 Hz, and time constants of 0.3 seconds (A and B) and 0.1 s (C). In the background activity, generalized slow waves are frequently observed during wakefulness, along with an alpha rhythm in the occipital lobe, as normal waking background is rarely seen. (B) In the EEG recorded during the interictal period, multifocal spike-and-wave patterns are observed during sleep (arrow). (C) Paroxysmal spikes are reduced, and a diffuse, irregular high-amplitude slow-wave background is observed during a cluster of epileptic spasms. PALE, post-transplant acute limbic encephalitis; vEEG, video electroencephalogram

No abnormalities were observed in the patient’s perinatal or developmental history. The patient had no family or medical history of convulsions. On neurological assessment, he could open his eyes and respond to calls but was unable to speak or follow commands; his Glasgow Coma Scale (GCS) score was 9 (E4V1M4). Blood tests revealed no abnormalities, and anti-glutamate receptor antibodies were negative, with absorbance values of 0.225 for GluN1-NT antibodies (normal value 0.300 ± 0.110) and 0.218 for GluN2B-NT antibodies (normal value 0.337 ± 0.133). On the video EEG (vEEG), generalized slow waves were frequently interspersed with background activity during wakefulness (Figure 3A). During the interictal period, multifocal spike wave activity was observed throughout the brain (Figure 3B). No rapid rhythms were observed during sleep. During a 24-hour period, approximately 10 episodes of seizures, characterized by brief contractions of the upper limbs, were recorded. During these seizures, EEG revealed high-amplitude generalized slow waves followed by attenuation (Figure 3C). Based on these findings, the seizures were diagnosed as epileptic spasms. We also observed tonic seizures lasting several seconds in the upper limbs, followed by epileptic spasms. On brain MRI 17 months after the onset of PALE, atrophy of the entire brain, mainly the right hippocampus, was observed (Figures 2D, 2E).

Based on the patient’s clinical findings and vEEG data, we diagnosed epileptic encephalopathy, and treatment with LEV (maximum dose of 42 mg/kg/day) was resumed. However, as no obvious therapeutic effects were observed, LCM (maximum dose of 6.7 mg/kg/day) was administered. The addition of lamotrigine (maximum dose of 3.9 mg/kg/day) and clobazam (maximum dose of 0.3 mg/kg/day) reduced the seizure frequency; however, a series of seizures continued to occur approximately twice a day, even more than one year following the onset of epilepsy.

Written informed consent for publication, including MRI findings, was obtained from the patient’s parents.

## Discussion

A handful of previous reports have focused on children who developed epileptic encephalopathy several months after HHV6 PALE, similar to the present case [4-6]. Five patients presented with multiple types of seizures, experienced developmental regression or cognitive decline, and showed hippocampal abnormalities on brain imaging. Although the number of reports is limited, the clinical symptoms, imaging findings, and disease course were similar, suggesting that the condition could be defined as epilepsy syndrome or epileptic encephalopathy following HHV6-related PALE (EE-PALE). The clinical characteristics of the five children with EE-PALE reported previously and the present case are summarized in Table 1. All patients were under 18 years of age at the onset of EE-PALE, three of the six patients were under four years of age, and no similar reports exist in adults. The sex ratio was five girls to one boy. The median time from the onset of HHV6 PALE to epilepsy was 11.5 months (range: 5-18 months). In the present case, the patient developed clinical seizures eight months after the onset of HHV6 PALE and was diagnosed 11 months later. Therefore, the disease course was consistent with that of the previously reported cases. All the patients exhibited developmental regression or cognitive decline. The three cases that developed in early childhood exhibited developmental regression, whereas those that developed in school-aged children demonstrated behavioral abnormalities, memory, and language impairments in addition to cognitive decline. On interictal EEG, all patients, including ours, demonstrated generalized slow waves. In this case, the predominant seizure type was epileptic spasms, which was also the initial seizure type in three of the cases. All the patients exhibited multiple seizure types, although tonic seizures were relatively common. However, a rapid rhythm during sleep was not observed in any of the cases; therefore, the EEG findings were different from those of typical Lennox-Gastaut syndrome. Additionally, atrophy of the right hippocampus was observed. Similarly, hippocampal sclerosis (HS) was observed on one or both sides in three of five previously reported cases. However, these seizure types differ from those observed in patients with medial temporal lobe epilepsy and HS. Regarding prognosis, as all patients had seizures for longer than one year, controlling seizures with drug therapy was predicted to be difficult. In case 5, adrenocorticotropic hormone therapy led to a temporary reduction in seizures; however, after a few months, the seizures returned and did not disappear [6]. In the present case, the frequency of seizures decreased due to combined therapy with multiple antiepileptic drugs; however, the seizures persisted for more than one year after the onset of epilepsy. Palliative surgical treatments including vagus nerve stimulation for seizure suppression are viable options.

**Table 1:** Summary of previously reported cases and the present case of EE-PALE ACTH, adrenocorticotropic hormone; CSF, cerebrospinal fluid; EEG, electroencephalogram; EE-PALE, epileptic encephalopathy following HHV6-related PALE; HHV6, human herpes virus 6; IVMP, intravenous methylprednisolone; IVIG, intravenous immunoglobulin; MRI, magnetic resonance imaging; N/A, not applicable; OCB, oligoclonal band; PALE, post-transplant acute limbic encephalitis

Case	1	2	3	4	5	6
Reference	Howell et al. [4]	Howell et al. [4]	Howell et al. [4]	Raspall-Chaure et al. [5]	Maizuru et al. [6]	This case
Gender	Male	Female	Male	Male	Male	Male
Age at time of transplant (year)	14	1	2	3	7	7
Time from transplant to onset of HHV6 PALE (day)	20-23			35	25	22
Time from onset of HHV6 PALE to onset of epilepsy (month)	18	14	11	5	5	11
Seizure type	Tonic and atonic	Tonic, atonic, atypical absence, and myoclonus	Tonic, atonic, and myoclonus	Epileptic spasm, tonic, and myoclonus	Epileptic spasm and tonic	Epileptic spasm and tonic
Coexisting neuropsychiatric symptoms	Cognitive decline	Developmental regression	Developmental regression	Developmental regression	Cognitive decline	Cognitive decline
Interictal EEG	Generalized slow spike-wave activity	Generalized slow spike-wave activity	Generalized slow spike-wave activity	Generalized and multifocal slow waves	Generalized slow spike-wave activity	Generalized slow waves, multifocal spike-wave activity
MRI finding	Hardening of the bilateral hippocampus	Swelling of the bilateral hippocampus	Hardening of the left hippocampus	Hardening of the right hippocampus	Atrophy of the bilateral hippocampus	Atrophy of the right hippocampus
OCB in the CSF	Negative	Positive	Negative	Positive	Negative	N/A
Autoantibodies	N/A	Negative	N/A	Negative	N/A	Negative
Immunomodulatory therapy	N/A	IVIG	N/A	IVMP and IVIG	ACTH	N/A
Outcome	Refractory seizures	Refractory seizures	Refractory seizures	Refractory seizures	Refractory seizures (briefly suppressed after ACTH)	Refractory seizures

EE-PALE may be caused by 1) secondary activation of the autoimmune system after an acute infection, 2) chronic inflammation after an acute infection, or 3) sequelae after limbic encephalitis. In all previously reported cases, reactivation of HHV6 was not observed at the onset of epilepsy, nor were any findings suggestive of chronic inflammation. The presence of oligoclonal bands (OCBs) in the cerebrospinal fluid of two of the five previously reported cases, along with reports of autoimmune epilepsy following simple herpes virus encephalitis, suggest that secondary activation of the autoimmune system may play a role in the pathogenesis of EE-PALE [8]. However, we were unable to find any reports on the detection of autoantibodies such as anti-glutamate receptor antibodies, which are associated with limbic encephalitis in patients with EE-PALE. However, these antibodies were not detected in the present case. Even in the two cases positive for OCBs in the cerebrospinal fluid, the effectiveness of immunomodulatory therapies, such as methylprednisolone pulse and intravenous immunoglobulin therapies, has not yet been demonstrated. In the present case, immunomodulatory therapy was difficult to administer due to the risk of recurrence.

In summary, EE-PALE is characterized by the following clinical features: 1) typical onset during childhood, 2) onset approximately one year (range: 5-18 months) after HHV6 PALE, 3) seizures of two or more types (e.g., tonic seizures, atonic seizures, and epileptic spasms), 4) cognitive decline or developmental regression, 5) interictal EEG displaying generalized slow waves but unlike typical Lennox-Gastaut syndrome, 6) MRI demonstrating sclerosis or atrophy of the hippocampus, and 7) ineffectiveness of antiepileptic drugs.

However, the exact pathogenesis remains unclear. Further research is needed to elucidate the pathogenesis of this disease and identify effective treatments. As EE-PALE is rare, this report may help explain its pathogenesis.

## Conclusions

This case provides additional evidence that EE-PALE is a distinct disease with uniform clinical features. In the present case, the patient developed epileptic encephalopathy following HHV6 PALE with diverse and intractable seizures, significant EEG abnormalities, hippocampal changes, and cognitive decline, as previously reported. Based on the present case and previous reports, we can state that these clinical signs are common symptoms of EE-PALE. Autoimmune mechanisms may also be involved in disease onset. We recommend considering EE-PALE when seizures and cognitive impairment appear several months after its onset. Further research is required to clarify the pathogenesis of this disease and develop effective treatments.
